# Whole-Genome Sequence of *Corynebacterium auriscanis* Strain CIP 106629 Isolated from a Dog with Bilateral Otitis from the United Kingdom

**DOI:** 10.1128/genomeA.00683-16

**Published:** 2016-08-11

**Authors:** Sandeep Tiwari, Syed Babar Jamal, Leticia Castro Oliveira, Dominique Clermont, Chantal Bizet, Diego Mariano, Paulo Vinicius Sanches Daltro de Carvalho, Flavia Souza, Felipe Luiz Pereira, Siomar de Castro Soares, Luis C. Guimarães, Fernanda Dorella, Alex Carvalho, Carlos Leal, Debmalya Barh, Henrique Figueiredo, Syed Shah Hassan, Vasco Azevedo, Artur Silva

**Affiliations:** a Institute of Biologic Sciences, Federal University of Minas Gerais, Belo Horizonte, Minas Gerais, Brazil; b Institut Pasteur, Unité de Prévention et Thérapies Moléculaires des Maladies Humaines, National Centre of Reference of Toxigenic Corynebacteria, Paris, France; c AQUACEN, National Reference Laboratory for Aquatic Animal Diseases, Ministry of Fisheries and Aquaculture, Federal University of Minas Gerais, Belo Horizonte, Minas Gerais, Brazil; d Department of Immunology, Microbiology and Parasitology, Institute of Biological Sciences and Natural Sciences, Federal University of Triângulo Mineiro (UFTM), Uberaba, Minas Gerais, Brazil; e Institute of Biological Sciences, Federal University of Pará, Belém, Pará, Brazil; f Centre for Genomics and Applied Gene Technology, Institute of Integrative Omics and Applied Biotechnology, Nonakuri, Purba Medinipur, West Bengal, India

## Abstract

In this work, we describe a set of features of *Corynebacterium auriscanis* CIP 106629 and details of the draft genome sequence and annotation. The genome comprises a 2.5-Mbp-long single circular genome with 1,797 protein-coding genes, 5 rRNA, 50 tRNA, and 403 pseudogenes, with a G+C content of 58.50%.

## GENOME ANNOUNCEMENT

The genus *Corynebacterium* contains many species that are pathogenic to humans and animals (1). Collins et al. (2) reported six coryneform-like isolates that originated from clinical specimens of bilateral otitis in dogs. They gave the name *Corynebacterium auriscanis* to one of the six coryneform-like isolates (3). Until 2008, *Corynebacterium auriscanis* was the only recognized animal pathogen, but a case reported in healthy human patient followed by a dog bite confirmed that this organism is a potential human pathogen and possibly a zoonotic carrier (3).

Here, we present the first draft genome sequence of *Corynebacterium auriscanis* CIP 106629 isolated from the clinical specimens in the United Kingdom. This bacterium is a Gram-positive, non-spore-forming, nonmotile, nonlipophilic, and typically club-shaped rod with appearance as single, in pairs, or in cluster cells. It is nonfermentative, nitrate reduction negative, and grows under aerobic conditions (4, 5). We determined the nucleotide sequence of the *C. auriscanis* CIP 106629 genome, isolated from a dog’s ear infection. Sequencing was performed by the National Reference Laboratory for Aquatic Animal Diseases, Ministry of Fisheries and Aquaculture, Federal University of Minas Gerais, Belo Horizonte, Minas Gerais, Brazil. Assembly and annotation were performed by the Laboratory of Cellular and Molecular Genetics, Federal University of Minas Gerais, Belo Horizonte, Minas Gerais, Brazil, and the Center of Genomics and Systems Biology, Federal University of Pará, Belém, Pará, Brazil. Automatic annotation was performed via the NCBI Prokaryotic Genome Annotation Pipeline (PGAP version 2.8) (http://www.ncbi.nlm.nih.gov/genome/annotation_prok/) (6).

The platform used for sequencing was the Ion Torrent Personal Genome Machine (PGM) system (Thermo Fisher), using a 200-bp fragment sequencing kit, according to the manufacturer’s protocols. The quality of the raw data was analyzed using the Web tool FastQC (http://www.bioinformatics.babraham.ac.uk/projects/fastqc). The reads with good quality were *de novo* assembled using the Mira version 3.9 software (7). The assembly produced 33 contigs, having a coverage of 78×, with *N*_50_ value for contig length of 241,167 bp.

The protein-coding genes (open reading frames [ORFs]) of the draft genome comprise a 2.5-Mbp-long single circular genome with 1,797 protein-coding genes, 5 rRNA, 50 tRNA, and 403 pseudogenes, with a G+C content of 58.50%.

### Nucleotide sequence accession numbers.

The *C. auriscanis* CIP 106629 whole-genome shotgun (WGS) project has the project accession no. JRVJ00000000. The version described in this paper is the first version and consists of sequences JRVJ00000000.1 to JRVJ00000000.33.
